# Predictors of reported alcohol intake during the first and second waves of the COVID-19 pandemic in Canada among middle-aged and older adults: results from the Canadian Longitudinal Study on Aging (CLSA)

**DOI:** 10.17269/s41997-022-00661-5

**Published:** 2022-07-11

**Authors:** Jacqueline M. McMillan, David B. Hogan, Chantelle Zimmer, Nazmul Sohel, Christina Wolfson, Susan Kirkland, Lauren E. Griffith, Nicole E. Basta, Parminder Raina, on behalf of the Canadian Longitudinal Study on Aging (CLSA) Team

**Affiliations:** 1 Division of Geriatric Medicine, Cumming School of Medicine, University of Calgary, 11th Floor, Rm 1104, South Tower, FMC, 1403 - 29th Street NW, Calgary, AB T2N 2T9 Canada; 2 Brenda Strafford Centre on Aging, O’Brien Institute for Public Health, University of Calgary, Calgary, Alberta Canada; 3 Department of Health Research Methods, Evidence, and Impact, McMaster University, Hamilton, Ontario Canada; 4 Research Institute of the McGill University Health Centre, Montreal, Quebec Canada; 5 Department of Medicine, McGill University, Montreal, Quebec Canada; 6 Department of Epidemiology and Biostatistics, McGill University, Montreal, Quebec Canada; 7 Department of Community Health & Epidemiology and Division of Geriatric Medicine, Dalhousie University, Halifax, Nova Scotia Canada; 8 Labarge Centre for Mobility in Aging, McMaster University, Hamilton, Ontario Canada; 9 McMaster Institute for Research on Aging, McMaster University, Hamilton, Ontario Canada

**Keywords:** COVID-19, CLSA, Alcohol use, Binge drinking, SARS-CoV-2, Older adults, COVID-19, ELCV, consommation d’alcool, hyperalcoolisation rapide, SRAS-CoV-2, personne âgée

## Abstract

**Objective:**

To examine proportions and predictors of change in alcohol intake and binge drinking during the first 2 waves of the COVID-19 pandemic among middle-aged and older participants in the Canadian Longitudinal Study on Aging (CLSA) COVID-19 Questionnaire Study.

**Methods:**

A total of 28,559 (67.2% of the potential sample) CLSA participants consented to the study with 24,114 completing the exit survey (fall 2020). Descriptive statistics and logistic regressions to examine predictors of change (increase or decrease) in alcohol intake and binge drinking were performed.

**Results:**

Among alcohol users, 26.3% reported a change in alcohol consumption during the first 10 months of the pandemic. Similar percentages increased (13.0%) or decreased (13.3%) consumption. In our mutually adjusted logistic regression model, odds of change in alcohol intake were greater for younger age, higher income, current cannabis smoker, positive screen for depression, anxiety, and loneliness. The magnitude of all associations for decreased intake was less than that of increased intake, and the directions were opposite for male sex and age. Predictors of current binge drinking (27.9% of alcohol users) included male sex, younger age, higher education and income, cannabis use, depression, and anxiety.

**Conclusion:**

Factors predictive of potentially worrisome alcohol use (i.e. increased intake, binge drinking) included younger age, sex, greater education and income, living alone, cannabis use, and worse mental health. Some of these factors were also associated with decreased intake, but the magnitudes of associations were smaller. This information may help direct screening efforts and interventions towards individuals at risk for problematic alcohol intake during the pandemic.

**Supplementary Information:**

The online version contains supplementary material available at 10.17269/s41997-022-00661-5.

## Résumé

**Objectif:**

Examiner les proportions et les prédicteurs des changements dans la consommation d’alcool et l’hyperalcoolisation rapide au cours des deux premières vagues de la pandémie de COVID-19 chez les personnes âgées et d’âge moyen ayant participé à l’étude par questionnaire sur la COVID-19 de l’Étude longitudinale canadienne sur le vieillissement (ELCV).

**Méthode:**

Un total de 28 559 participants de l’ELCV (67,2 % de l’échantillon potentiel) ont consenti à l’étude sur la COVID-19, et 24 114 ont répondu à l’enquête à la sortie (automne 2020). Nous avons procédé par statistique descriptive et par régression logistique pour examiner les prédicteurs des changements (augmentation ou diminution) dans la consommation d’alcool et l’hyperalcoolisation rapide.

**Résultats:**

Chez les consommateurs d’alcool, 26,3 % ont déclaré un changement de leur consommation d’alcool au cours des 10 premiers mois de la pandémie. Un pourcentage semblable de consommateurs d’alcool avaient accru (13 %) ou diminué (13,3 %) leur consommation. Dans notre modèle de régression logistique mutuellement ajusté, la probabilité de changement dans la consommation d’alcool était plus élevée chez les répondants plus jeunes, les répondants au revenu élevé, les fumeurs de cannabis actuels et les répondants ayant fait état de dépression, d’anxiété ou de solitude. Les associations avec la diminution de la consommation étaient moins significatives qu’avec l’augmentation de la consommation, et elles allaient dans le sens opposé pour ce qui est du sexe masculin et de l’âge. Les prédicteurs de l’hyperalcoolisation rapide actuelle (27,9 % des consommateurs d’alcool) étaient le sexe masculin, l’âge plus jeune, l’instruction et le revenu élevés, la consommation de cannabis, la dépression et l’anxiété.

**Conclusion:**

Les facteurs pouvant prédire une consommation d’alcool potentiellement inquiétante (c.-à-d. consommation accrue, hyperalcoolisation rapide) étaient l’âge plus jeune, le sexe, l’instruction et le revenu élevés, le fait de vivre seul, la consommation de cannabis et la moins bonne santé mentale. Certains de ces facteurs étaient aussi associés à une consommation réduite, mais ces associations étaient moins significatives. Ces informations pourraient orienter les efforts de dépistage et les interventions auprès des personnes à risque de consommation problématique d’alcool durant la pandémie.

## Introduction

Nearly one in five Canadians ≥ 18 years of age responding to a web-based survey conducted in January 2021 reported drinking more alcohol during the early months of the coronavirus disease 2019 (COVID-19) pandemic (Statistics Canada, 2021a). Of these respondents, about one fifth (18%) reported binge drinking (defined by the authors of this particular study as drinking 5 or more drinks on the days they consumed alcohol) compared to 11% pre-pandemic with ~ 36% of binge drinkers consuming this amount during the previous week (Statistics Canada, 2021a). Lack of a regular schedule, boredom, and stress were the three most common reasons cited for increased intake (Nanos, 2020), which were identical to the 22% of Canadians ≥ 65 years who reported greater cannabis use over the same period (Statistics Canada, 2021a).

Alcohol intake remained unchanged for 54–70% of participants in three early Canadian COVID-19 studies that included questions on alcohol consumption (Statistics Canada, 2021a; Nanos, 2020; Shield et al., 2020). When change occurred, it tended to be more often increased (18–23% of respondents) than decreased (12–22%) intake (Statistics Canada, 2021a; Nanos, 2020; Shield et al., 2020). Characteristics associated with changes in intake included age, employment status, living situation, and household income (Shield et al., 2020). When considered separately, factors associated with increased intake were loneliness, social isolation, anxiety, depression, disruption to one’s regular schedule, less clear delineation between work and leisure time, drinking alcohol with meals at home, and boredom (Statistics Canada, 2021a; Nanos, 2020; Shield et al., 2020). Factors associated with decreased intake included fewer opportunities for socialization, lifestyle choices (e.g. weight control, optimization of health, dislike of alcohol effects), cost, and additional responsibilities (e.g. providing care) (Statistics Canada, 2021a; Nanos, 2020; Shield et al., 2020).

As moderate and high levels of alcohol use can pose health concerns (Lee & Walter, 2017), increases in consumption related to the pandemic could adversely affect health. In Alberta, hospitalizations for alcoholic hepatitis rose from 11.6 to 22.1 per 10,000 admissions between March and September 2020 (during the first COVID-19 wave) (Black, 2021). Canadian guidelines for alcohol use disorder among older adults advise no more than 1 standard drink per day (no more than 5 per week) for women ≥ 65 years, and no more than 1–2 standard drinks per day (no more than 7 per week) for men ≥ 65 years (Butt et al., 2020). These guidelines do not provide a definition of binge drinking, a common pattern for excessive alcohol intake. Binge drinking in people ≥ 65 years has been defined elsewhere as 4+ drinks for women and 5+ drinks for men on one occasion (Al-Rousan et al., 2022; Han et al., 2017). It is associated with adverse health outcomes, including liver disease and premature death (Stahre et al., 2014), and may have increased during the pandemic.

Beliefs around alcohol use based on the integrated behaviour model (Montano & Kaspryzk, 2008) can be categorized into four: emotional beliefs, normative beliefs, behavioural beliefs, and beliefs around control (Leclair et al., 2020). Emotional beliefs are related to the emotional response one expects as a result of drinking (e.g. joy or sadness) (Leclair et al., 2020). Normative beliefs are related to expectations and are influenced by age, gender, and social support among other factors (Leclair et al., 2020). Behavioural beliefs are related to the association between alcohol and pleasure, socializing, expectation of relaxing effects, consumption as a method of managing emotions, and expected health benefits (Leclair et al., 2020). Finally, beliefs about control can influence alcohol consumption. Studies have shown that individuals with a strong sense of control consume less alcohol (Leclair et al., 2020).

The aim of this study was to examine the proportion and factors associated with changes in alcohol intake and binge drinking during the first 10 months (corresponding to the first 2 waves) of the COVID-19 pandemic among middle-aged and older participants in the CLSA COVID-19 Questionnaire Study. The selection of explanatory factors we examined was informed by the belief framework, the results of prior studies that reported on factors associated with changes in alcohol intake during the COVID-19 pandemic, and their availability within the Canadian Longitudinal Study on Aging (CLSA) dataset. As the effect of cannabis use on alcohol intake has not been explored in prior Canadian pandemic studies, we included this characteristic in our search for associated factors.

## Methods

### Study setting and population

The CLSA is a nationally generalizable longitudinal study of 51,338 Canadian residents 45–85 years of age at the time of their enrolment (2011–2015). Comprehensive biological, medical, psychological, social, lifestyle, and economic data are collected on study participants every 3 years. At the first follow-up (2015–2018), retention was 95% with data obtained on 48,893 individuals (Raina et al., 2009; Raina et al., 2019).

**Fig. 1:**
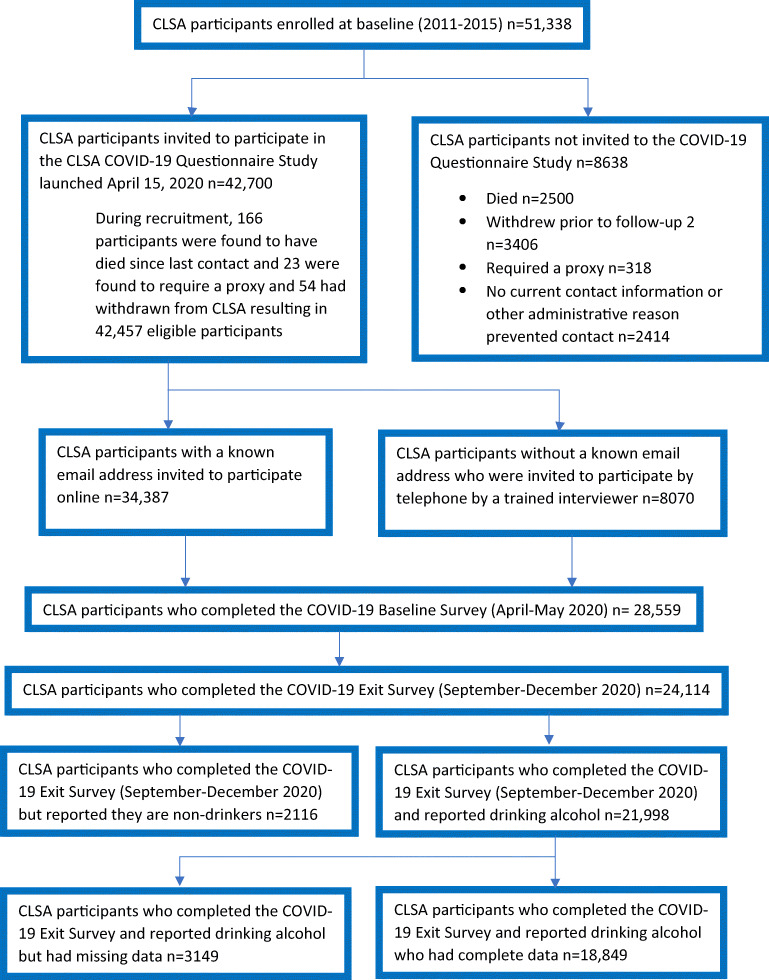
CLSA COVID-19 Questionnaire Study participant flow diagram

CLSA participants were contacted in April 2020 and were invited to participate in the COVID-19 Questionnaire Study. Inclusion criteria were active participation in the CLSA, ability to personally provide requested data, and updated contact information. Of the 42,457 eligible participants, 28,559 (67.3%) agreed to participate and completed the baseline survey. Of this group, 24,114 (84.4%) completed the exit survey in the fall of 2020. In this latter group, 21,998 (91.2%) reported drinking alcohol. Those consuming alcohol who also provided complete data were retained (*n* = 18,849; 85.7%) in the complete case analysis. Further details of the COVID-19 Questionnaire Study are available elsewhere (Raina et al., 2021) while Fig. 1 shows participant flow.

### Data sources

Data from the CLSA baseline (2011–2015) and CLSA follow-up 1 (2015–2018) as well as the CLSA COVID-19 Questionnaire Study baseline (April–May 2020) and exit (September–December 2020) surveys were utilized for these analyses.

### Outcome variables

Change in alcohol intake and presence of binge drinking were assessed in the COVID-19 exit survey. Participants were asked, “Have you ever drank alcohol?” Possible responses were yes, no, don’t know/no answer, or refused. If participants answered yes, they were asked whether consumption had increased, decreased, or stayed the same since March 1, 2020. One binary outcome variable was created: change in alcohol intake versus no change, and one three-level outcome variable was created: increase, decrease, and stayed the same.

Participants who drank alcohol were also asked, “About how often since March 1, 2020 would you say you had five [four for women] or more drinks at the same sitting or occasion?” Possible responses were: almost every day (6–7 times a week), 4–5 times a week, 2–3 times a week, once a week, 2–3 times a month, about once a month, less than once a month, never, don’t know/no answer, or prefer not to answer. We defined regular binge drinking as occurring if the frequency was once or more per month and occasional as less often than monthly.

### Explanatory variables

- *Sociodemographic variables*

Birth sex (male, female), age group (< 65, 65–74, ≥ 75), educational attainment (less than secondary school graduation, secondary school graduation, some post-secondary education, post-secondary degree), annual household income (in Canadian dollars; < $20,000, $20,000 to $49,999, $50,000 to $99,999, $100,000 to $149,999, $150,000 or greater), province of residence, and household composition (one [living alone], two or more people living in the same household) were examined for their associations with alcohol use. Sex and education data were extracted from the CLSA baseline assessment; annual household income from follow-up 1; province of residence and household composition from the COVID-19 baseline survey; and, age group from the COVID-19 exit survey.

(b) *Mental health variables*

The presence of depressive symptoms was assessed using the Center for Epidemiologic Studies Short Depression Scale (CESD-10) (Andresen et al., 1994). Scores range from 0 to 30 with a value of ≥ 10 used to determine the presence of depression (Andresen et al., 1994). Anxiety was evaluated by the Generalized Anxiety Disorder scale (GAD-7) (Spitzer et al., 2006). Scores of 0–4 were classified as no/minimal, 5–9 as mild, 10–14 as moderate, and 15–21 as severe anxiety (Spitzer et al., 2006). Scores on the 3-item UCLA Loneliness Scale (Russell, 1996) range from 3 to 9 with a cut-off of ≥ 6 used to identify loneliness (Steptoe et al., 2013) The personal impact of the pandemic on participants was determined using the question, “Taking everything about COVID-19 into account, how would you describe the consequences of COVID-19 on you and your household?” Possible responses were: very negative, negative, no effect, positive, very positive, don’t know/no answer, or prefer not to answer (Cao-Lei et al., 2016). All data were extracted from the COVID-19 exit survey.

(c) *Cannabis use variable*

Cannabis use data were obtained from the COVID-19 exit survey. Participants were asked, “In your lifetime, have you smoked marijuana/cannabis for one month or longer?” Possible responses included: yes, no, don’t know/no answer, or prefer not to answer. Participants who responded yes were asked, “Do you currently smoke marijuana/cannabis?” Cannabis use was then categorized as current, previous, or never.

### Statistical analysis

Descriptive analyses were performed to determine the proportion with change in alcohol intake and binge drinking status as well as examining sociodemographic variables (age, sex, education, income, and province) and select predictors (living alone, cannabis use, screen for depression, anxiety severity, loneliness, and consequences of COVID-19). Means and standard deviations are reported for continuous variables and frequencies and percentages for categorical variables. The association of self-reported change in alcohol intake (either increase or decrease versus no change) during the first 10 months of the COVID-19 pandemic and binge drinking status were then assessed with sociodemographic variables and select predictors. First, any change in alcohol intake was assessed with sociodemographic variables (core model), and adjusted odds ratios (aOR) were presented with 95% confidence intervals (95% CI). We then investigated the association of change in alcohol intake separately for increased and decreased intake with no change to assess the magnitude and direction of the association(s), employing multinomial logistic regression. We dichotomized binge drinking (yes/no) and assessed its association with the same set of covariates. Each predictor was added separately to the core model to see the association with the outcomes. All sociodemographic variables and predictors were included in the final model.

We employed the SAS proc Logistic procedure for estimating odds ratios and used “glogit” link function for multinomial logistic regression. Approximately 14% of participants were missing data (either one of the sociodemographic variables or predictors). We investigated missing data patterns and employed multiple imputation for sensitivity analysis. We employed multivariate models using conditional distribution where every variable is imputed conditional on all other variables. Proc MI procedure was used to impute missing data. The number of imputations was set to 10 and the fully conditional specification regression method was used. All analyses were conducted using SAS version 9.4 (SAS/STAT Software. 2021).

### Ethics

The COVID-19 Questionnaire Study, and the CLSA baseline and follow-up studies, have been approved by the research ethics boards at all collaborating Canadian institutions. The present study was approved by the University of Calgary Research Ethics Board *REB21-0972.*

## Results

Of the 21,998 participants who reported that they drank alcohol, 3149 participants had missing data for one or more covariates (education = 33, income = 1275, living alone = 361, cannabis use = 218, depression = 155, anxiety = 490, loneliness = 165, and consequences of COVID = 452). Missing data was greater in older women with lower education, and those who lived alone, never smoked marijuana/cannabis, had a positive screen for depression, had anxiety, and were lonely (Supplementary Table 1). As can also be seen, missing data varied across income levels, provinces, and consequences of COVID-19.

In complete case analysis, the majority (*n* = 13,901, 73.7%) of participants reported no change in alcohol intake since March 1, 2020. Similar proportions reported increased (*n* = 2450, 13%) or decreased (*n* = 2498, 13.3%) consumption (Table 1).

**Table 1:** Characteristics of the sociodemographic variables and selected predictors of the study population

	Change in alcohol consumption since March 1, 2020				Binge drinking			
	Increased	Decreased	Stayed the same	Total	Not at all	Occasional	Regular	Total
Age								
Mean	64.68	69.78	69.51	68.91	70.28	65.92	65.01	68.91
SD	7.79	9.50	9.29	9.28	9.40	8.15	7.81	9.29
Min	51	51	50	50	50	51	51	50
Max	91	93	96	96	96	95	93	96
Range	40	42	46	46	46	44	42	46
Total	2450	2498	13,901	18,849	13,551	2106	3136	18,793
	13.0	13.3	73.7	100.0	72.1	11.2	16.7	100.0
Sex at baseline								
Male	1119	1309	6892	9320	6481	1069	1744	9294
	12.0	14.0	73.9	100.0	69.7	11.5	18.8	100.0
Female	1331	1189	7009	9529	7070	1037	1392	9499
	14.0	12.5	73.6	100.0	74.4	10.9	14.7	100.0
3-level age group at COVID exit								
< 65	1275	796	4552	6623	4009	976	1625	6610
	19.3	12.0	68.7	100.0	60.7	14.8	24.6	100.0
65–74	904	949	5264	7117	5143	801	1146	7090
	12.7	13.3	74.0	100.0	72.5	11.3	16.2	100.0
75+	271	753	4085	5109	4399	329	365	5093
	5.3	14.7	80.0	100.0	86.4	6.5	7.2	100.0
Province of residence at COVID								
Newfoundland	91	139	840	1070	731	141	194	1066
	8.5	13.0	78.5	100.0	68.6	13.2	18.2	100.0
Prince Edward Island	24	26	237	287	213	31	43	287
	8.4	9.1	82.6	100.0	74.2	10.8	15.0	100.0
Nova Scotia	201	202	1278	1681	1148	203	326	1677
	12.0	12.0	76.0	100.0	68.5	12.1	19.4	100.0
New Brunswick	26	45	287	358	277	28	52	357
	7.3	12.6	80.2	100.0	77.6	7.8	14.6	100.0
Quebec	413	415	2530	3358	2246	413	688	3347
	12.3	12.4	75.3	100.0	67.1	12.3	20.6	100.0
Ontario	645	643	3171	4459	3282	462	702	4446
	14.5	14.4	71.1	100.0	73.8	10.4	15.8	100.0
Manitoba	204	242	1186	1632	1195	174	262	1631
	12.5	14.8	72.7	100.0	73.3	10.7	16.1	100.0
Saskatchewan	27	56	328	411	328	35	46	409
	6.6	13.6	79.8	100.0	80.2	8.6	11.2	100.0
Alberta	235	239	1360	1834	1365	208	252	1825
	12.8	13.0	74.2	100.0	74.8	11.4	13.8	100.0
British Columbia	584	491	2684	3759	2766	411	571	3748
	15.5	13.1	71.4	100.0	73.8	11.0	15.2	100.0
4-level education at baseline								
Less than secondary school	31	85	606	722	588	57	73	718
	4.3	11.8	83.9	100.0	81.9	7.9	10.2	100.0
Secondary school graduation	169	234	1334	1737	1238	190	301	1729
	9.7	13.5	76.8	100.0	71.6	11.0	17.4	100.0
Some post-secondary education	142	215	1001	1358	970	141	243	1354
	10.5	15.8	73.7	100.0	71.6	10.4	17.9	100.0
Post-secondary degree/diploma	2108	1964	10960	15032	10755	1718	2519	14992
	14.0	13.1	72.9	100.0	71.7	11.5	16.8	100.0
5-level annual household income at follow-up								
Less than $20,000	44	80	496	620	520	44	55	619
	7.1	12.9	80.0	100.0	84.0	7.1	8.9	100.0
$20,000–$49,999	287	521	2890	3698	3017	296	365	3678
	7.8	14.1	78.2	100.0	82.0	8.0	9.9	100.0
$50,000–$99,999	786	968	5383	7137	5255	746	1119	7120
	11.0	13.6	75.4	100.0	73.8	10.5	15.7	100.0
$100,000–$149,999	650	505	2891	4046	2746	513	776	4035
	16.1	12.5	71.5	100.0	68.1	12.7	19.2	100.0
$150,000 or more	683	424	2241	3348	2013	507	821	3341
	20.4	12.7	66.9	100.0	60.3	15.2	24.6	100.0
Number living in household at COVID baseline								
One (living alone)	480	658	3296	4434	3430	382	603	4415
	10.8	14.8	74.3	100.0	77.7	8.7	13.7	100.0
Two	1970	1840	10,605	14,415	10,121	1724	2533	14,378
	13.7	12.8	73.6	100.0	70.4	12.0	17.6	100.0
Smoked cannabis 1 month or longer								
Never	1853	2081	11,932	15,866	11,909	1670	2241	15,820
	11.7	13.1	75.2	100.0	75.3	10.6	14.2	100.0
Previous	346	254	1300	1900	1152	266	477	1895
	18.2	13.4	68.4	100.0	60.8	14.0	25.2	100.0
Current	251	163	669	1083	490	170	418	1078
	23.2	15.1	61.8	100.0	45.5	15.8	38.8	100.0
CES-D 10: screen for depression result								
Negative screen for depression	1633	1906	11,334	14,873	10,815	1660	2357	14,832
	11.0	12.8	76.2	100.0	72.9	11.2	15.9	100.0
Positive screen for depression	817	592	2567	3976	2736	446	779	3961
	20.5	14.9	64.6	100.0	69.1	11.3	19.7	100.0
GAD-7 anxiety severity classification								
No/minimal anxiety	1602	1890	11,131	14,623	10679	1600	2299	14,578
	11.0	12.9	76.1	100.0	73.3	11.0	15.8	100.0
Mild anxiety	620	466	2225	3311	2270	407	625	3302
	18.7	14.1	67.2	100.0	68.7	12.3	18.9	100.0
Moderate anxiety	163	103	407	673	440	77	156	673
	24.2	15.3	60.5	100.0	65.4	11.4	23.2	100.0
Severe anxiety	65	39	138	242	162	22	56	240
	26.9	16.1	57.0	100.0	67.5	9.2	23.3	100.0
UCLA loneliness scale—total score								
Negative	1769	1911	11,135	14,815	10,600	1700	2471	14771
	11.9	12.9	75.2	100.0	71.8	11.5	16.7	100.0
Positive	681	587	2766	4034	2951	406	665	4022
	16.9	14.6	68.6	100.0	73.4	10.1	16.5	100.0
Consequences of COVID-19 on self and household								
Negative	1806	1660	8495	11,961	8600	1354	1978	11,932
	15.1	13.9	71.0	100.0	72.1	11.3	16.6	100.0
No effect	469	648	4538	5655	4086	605	942	5633
	8.3	11.5	80.2	100.0	72.5	10.7	16.7	100.0
Positive	175	190	868	1233	865	147	216	1228
	14.2	15.4	70.4	100.0	70.4	12.0	17.6	100.0

### Predictors of change (increase or decrease) in alcohol intake compared to no change

As shown in Table 2, there was overall no difference by sex in the odds of change in alcohol intake since the start of the pandemic. Individuals < 65 years and 65–74 years had higher odds of reporting a change compared to ≥ 75 years, and all education levels had greater odds than those with less than high school graduation. All provinces had lower odds of reporting a change compared to Ontario. Odds of reporting a change in alcohol intake increased with higher income, anxiety severity, living alone, previous or current marijuana/cannabis smoking, and a positive depression screen. There was a non-significant trend towards greater odds of change among individuals who were lonely. Individuals who reported negative/very negative or positive/very positive consequences of COVID-19 for themselves and their household had higher odds of reporting a change in alcohol intake than those who reported no effect.

**Table 2:** Association of selected predictors with changes in alcohol intake (any changes and either increased or decreased) compared to no changes, mutually adjusted model presented with odds ratios (OR) along with 95% confidence interval (95% CI) in CLSA participants who completed the CLSA COVID-19 exit survey (fall 2020)

	Any change	Increased	Decreased
	OR (95% CI)	OR (95% CI)	OR (95% CI)
Sex at baseline			
Male	1.02 (0.95, 1.09)	0.88 (0.80, 0.96)	1.17 (1.07, 1.28)
Female	Ref		
3-level age group at COVID exit			
< 65	1.44 (1.31, 1.59)	2.84 (2.45, 3.30)	0.90 (0.80, 1.02)
65–74	1.27 (1.16, 1.39)	2.17 (1.88, 2.52)	0.96 (0.86, 1.07)
75+	Ref		
Province of residence at COVID			
Newfoundland	0.71 (0.60, 0.84)	0.55 (0.44, 0.70)	0.85 (0.70, 1.04)
Prince Edward Island	0.60 (0.44, 0.82)	0.62 (0.40, 0.96)	0.58 (0.38, 0.88)
Nova Scotia	0.80 (0.70, 0.92)	0.81 (0.68, 0.97)	0.79 (0.67, 0.94)
New Brunswick	0.68 (0.52, 0.90)	0.53 (0.35, 0.81)	0.81 (0.58, 1.13)
Quebec	0.92 (0.83, 1.03)	0.99 (0.86, 1.14)	0.87 (0.75, 0.99)
Ontario	Ref		
Manitoba	0.92 (0.81, 1.05)	0.83 (0.69, 0.99)	1.00 (0.85, 1.18)
Saskatchewan	0.64 (0.49, 0.82)	0.40 (0.27, 0.61)	0.86 (0.64, 1.15)
Alberta	0.83 (0.74, 0.94)	0.80 (0.68, 0.95)	0.86 (0.73, 1.01)
British Columbia	0.98 (0.89, 1.08)	1.07 (0.94, 1.22)	0.89 (0.78, 1.01)
4-level education at baseline			
Less than secondary school	Ref		
Secondary school graduation	1.35 (1.07, 1.70)	1.71 (1.14, 2.56)	1.27 (0.97, 1.66)
Some post-secondary education	1.47 (1.16, 1.87)	1.67 (1.10, 2.53)	1.49 (1.13, 1.96)
Post-secondary degree/diploma	1.46 (1.19, 1.81)	2.07 (1.42, 3.02)	1.26 (0.99, 1.60)
5-level annual household income at follow-up			
Less than $20,000	Ref		
$20,000–$49,999	1.26 (1.01, 1.57)	1.36 (0.96, 1.92)	1.18 (0.91, 1.53)
$50,000–$99,999	1.46 (1.17, 1.81)	1.90 (1.36, 2.66)	1.22 (0.94, 1.57)
$100,000–$149,999	1.69 (1.35, 2.12)	2.59 (1.84, 3.64)	1.20 (0.92, 1.58)
$150,000 or more	2.02 (1.61, 2.54)	3.27 (2.31, 4.62)	1.32 (1.00, 1.74)
Number living in household at COVID baseline			
One (living alone)	1.18 (1.08, 1.29)	1.16 (1.03, 1.31)	1.21 (1.08, 1.35)
Two or more	Ref		
Smoked cannabis 1 month or longer			
Never	Ref		
Previous	1.23 (1.10, 1.36)	1.35 (1.18, 1.54)	1.11 (0.96, 1.28)
Current	1.62 (1.42, 1.85)	1.91 (1.62, 2.24)	1.36 (1.13, 1.63)
CES-D 10: screen for depression result			
Negative screen for depression	Ref		
Positive screen for depression	1.41 (1.28, 1.56)	1.66 (1.46, 1.90)	1.20 (1.05, 1.37)
GAD-7 anxiety severity classification			
No/minimal anxiety	Ref		
Mild anxiety	1.19 (1.08, 1.31)	1.29 (1.13, 1.46)	1.10 (0.97, 1.25)
Moderate anxiety	1.39 (1.16, 1.67)	1.50 (1.20, 1.87)	1.25 (0.98, 1.60)
Severe anxiety	1.47 (1.12, 1.94)	1.53 (1.10, 2.12)	1.34 (0.92, 1.96)
UCLA loneliness scale—total score			
Negative	Ref		
Positive	1.07 (0.98, 1.17)	1.10 (0.98, 1.23)	1.05 (0.94, 1.18)
Consequences of COVID-19 on self and household			
Negative	1.42 (1.31, 1.54)	1.63 (1.45, 1.83)	1.27 (1.15, 1.40)
No effect	Ref		
Positive	1.64 (1.42, 1.88)	1.75 (1.44, 2.12)	1.57 (1.31, 1.87)

### Predictors of increased alcohol intake

Please see Table 2 for details. Males were less likely than females to report an increase in alcohol intake. Individuals < 65 and those 65–74 years of age had 2+-fold greater odds of increased intake compared to ≥ 75-year-olds. Having a post-secondary degree or diploma was likewise associated with higher odds of increased alcohol intake compared to less than high school education. Higher annual household income was associated with greater odds of increased intake. The odds were 1.4, 1.9, 2.6, and 3.3 times higher for incomes of $20,000 to $49,999, $50,000 to $99,999, $100,000 to $149,999, and > $150,000 respectively. Living alone was associated with marginally higher odds of increased alcohol intake. Previous and current marijuana/cannabis use were associated with 1.4 times and 1.9 times higher odds, respectively, of increased intake than not using. The magnitude of this effect was greater in females with current marijuana/cannabis users having 2.4 times greater odds of increased intake (Supplementary Tables 3a and 3b).

A positive screen for depression was associated with 1.7 times greater odds of increased intake and anxiety with 1.3 to 1.5 times greater odds (depending on anxiety severity) of increased intake compared to no/minimal anxiety. Individuals who reported negative/very negative or positive/very positive consequences of COVID-19 for themselves and their household had 1.6- and 1.7-fold higher odds, respectively, of increased alcohol intake.

### Predictors of decreased alcohol intake

Males were more likely to report decreased alcohol intake since the start of the pandemic, compared to females. Having some post-secondary education was associated with 1.5 times greater odds of decreased intake than less than high school education. An annual household income > $150,000 had 1.3-fold higher odds of decreased intake than one < $20,000. Living alone was associated with 1.2 times greater odds of decreased alcohol intake compared to living with others. Current cannabis use had 1.4 times greater odds of decreased alcohol intake than never smoking. This association was slightly greater in females compared to males.

A positive depression screen increased the odds of decreased intake by 1.2 times in females but was non-statistically significant in males. Both negative/very negative and positive/very positive consequences of COVID-19 on the participant and their household were associated with 1.3 and 1.6 times greater odds, respectively, of decreased alcohol intake.

### Binge drinking

Binge drinking data were available for 18,793 participants. Overall, 11.2% met criteria for occasional and 16.7% met criteria for regular binge drinking (Table 1). Of those who had increased their alcohol intake since the start of the pandemic, 46.2% met criteria for regular binge drinking and 13.6% for occasional binge drinking, and 40.2% did not meet criteria for binge drinking.

### Predictors of binge drinking

Men were more likely to report binge drinking than females. Individuals < 65 years and those 65–74 years were more likely to report binge drinking than those ≥ 75 years (Table 3).

**Table 3:** Association of selected predictors with binge drinking status presented with odds ratios (OR) along with 95% confidence interval (95% CI) in CLSA participants who completed the CLSA COVID-19 exit survey (fall 2020). Mutually adjusted model presented for both sexes and stratified by sex

	Both sexes	Male	Female
	OR (95% CI)	OR (95% CI)	OR (95% CI)
Sex at baseline			
Male	1.22 (1.13, 1.30)		
Female	Ref		
3-level age group at COVID exit			
< 65	2.92 (2.63, 3.23)	2.75 (2.40, 3.15)	3.21 (2.73, 3.77)
65–74	2.03 (1.83, 2.24)	1.89 (1.66, 2.16)	2.25 (1.92, 2.64)
75+	Ref		
Province of residence at COVID			
Newfoundland	1.33 (1.14, 1.55)	1.34 (1.09, 1.65)	1.30 (1.04, 1.63)
Prince Edward Island	1.09 (0.82, 1.46)	0.75 (0.49, 1.13)	1.63 (1.10, 2.41)
Nova Scotia	1.32 (1.16, 1.50)	1.30 (1.09, 1.55)	1.32 (1.09, 1.60)
New Brunswick	0.85 (0.65, 1.12)	0.83 (0.58, 1.19)	0.87 (0.58, 1.31)
Quebec	1.62 (1.45, 1.80)	1.46 (1.26, 1.69)	1.79 (1.54, 2.09)
Ontario	Ref		
Manitoba	1.04 (0.91, 1.19)	1.05 (0.87, 1.26)	1.04 (0.86, 1.26)
Saskatchewan	0.69 (0.53, 0.90)	0.64 (0.44, 0.94)	0.75 (0.52, 1.08)
Alberta	0.92 (0.81, 1.05)	0.92 (0.77, 1.10)	0.93 (0.77, 1.13)
British Columbia	0.99 (0.89, 1.10)	0.96 (0.83, 1.11)	1.02 (0.88, 1.19)
4-level education at baseline			
Less than secondary school	Ref		
Secondary school graduation	1.26 (1.01, 1.58)	1.19 (0.87, 1.62)	1.36 (0.97, 1.91)
Some post-secondary education	1.27 (1.00, 1.60)	1.17 (0.85, 1.62)	1.39 (0.98, 1.97)
Post-secondary degree/diploma	1.00 (0.81, 1.22)	0.87 (0.66, 1.15)	1.15 (0.85, 1.57)
5-level annual household income at follow-up			
Less than $20,000	Ref		
$20,000–$49,999	1.41 (1.11, 1.80)	1.33 (0.90, 1.97)	1.48 (1.08, 2.02)
$50,000–$99,999	2.19 (1.73, 2.78)	2.23 (1.53, 3.26)	2.16 (1.58, 2.94)
$100,000–$149,999	2.61 (2.04, 3.34)	2.49 (1.69, 3.66)	2.80 (2.02, 3.87)
$150,000 or more	3.42 (2.67, 4.39)	3.57 (2.42, 5.26)	3.25 (2.33, 4.53)
Number living in household at COVID baseline			
One (living alone)	1.08 (0.99, 1.19)	1.10 (0.96, 1.27)	1.09 (0.96, 1.24)
Two or more	Ref		
Smoked cannabis 1 month or longer			
Never	Ref		
Previous	1.52 (1.37, 1.69)	1.46 (1.27, 1.67)	1.64 (1.39, 1.93)
Current	2.93 (2.57, 3.34)	2.77 (2.34, 3.27)	3.26 (2.62, 4.05)
CES-D 10: screen for depression result			
Negative screen for depression	Ref		
Positive screen for depression	1.24 (1.11, 1.38)	1.16 (0.99, 1.36)	1.31 (1.13, 1.51)
GAD-7 anxiety severity classification			
No/minimal anxiety	Ref		
Mild anxiety	1.14 (1.03, 1.26)	1.10 (0.95, 1.28)	1.17 (1.02, 1.34)
Moderate anxiety	1.24 (1.02, 1.50)	1.19 (0.88, 1.61)	1.28 (1.00, 1.64)
Severe anxiety	1.06 (0.79, 1.43)	1.14 (0.70, 1.87)	1.01 (0.69, 1.48)
UCLA loneliness scale—total score			
Negative	Ref		
Positive	0.86 (0.79, 0.95)	0.89 (0.78, 1.03)	0.84 (0.74, 0.95)
Consequences of COVID-19 on self and household			
Negative	0.96 (0.89, 1.04)	0.96 (0.87, 1.07)	0.97 (0.86, 1.08)
No effect	Ref		
Positive	1.04 (0.90, 1.20)	0.98 (0.79, 1.22)	1.09 (0.90, 1.32)

Participants in Newfoundland, Nova Scotia, and Quebec and females (but not males) in PEI had greater odds of binge drinking than those living in Ontario, while participants from Saskatchewan had lower odds (Table 3). Higher annual household income predicted binge drinking (Table 3). Previous cannabis use was associated with 1.5 times greater odds and current cannabis use with 2.9 times greater odds of binge drinking. The magnitude of effect was larger for current female cannabis users than seen in males.

A positive depression screen increased the odds of binge drinking by 1.3-fold in females, but was not significant in males. The association between anxiety and binge drinking was only significant in females as well. Mild anxiety increased the odds of binge drinking in females by 1.2 times and moderate anxiety by 2.3 times. Severe anxiety and participants’ views of the consequences of COVID-19 on themselves and their households were not significantly associated with binge drinking, though loneliness in females was associated with decreased odds (Table 3).

### Sensitivity analysis

We created 10 copies of the dataset with missing values replaced with imputed values based on observed data. We then reproduced Tables 2 and 3, which can be seen as Supplementary Tables 4 and 5. Estimates were similar in most cases, with the same magnitude and direction retained in the imputed dataset that we observed in the complete case analysis.

## Discussion

The COVID-19 pandemic has resulted in major disruptions to everyday life. Both the academic and non-academic literature have reported increased alcohol sales and intake during this period (Benzie, 2020; Guignard et al., 2021; Jackson et al., 2021; Nanos, 2020; Reynolds et al., 2021; Shield et al., 2020; Vanderbruggen et al., 2020; Zussman, 2020). As noted previously, individuals may consume alcohol because of various beliefs. There are other proposed frameworks for understanding alcohol use. A motivational model of alcohol use asserts that the desire to drink arises from one’s expectations of how alcohol will make the person feel (Cox & Klinger, 1988). People may also drink alcohol for enhancement (excitement) and to socialize (celebrate), cope, and conform (Kuntsche & Callinan, 2018). Whatever the drivers might be, of concern is the known associations between moderate and high levels of alcohol use and adverse health outcomes (Black, 2021; Lee & Walter, 2017), aggression, violence and domestic abuse (Sontate et al., 2021), and impaired driving (Canadian Centre on Substance Use and Addiction, 2021a). Discovering one’s motivation to drink alcohol may help guide strategies to reduce harmful drinking behaviours.

We examined the proportion and factors associated with changes in alcohol intake and binge drinking during the first 10 months of the COVID-19 pandemic in middle-aged and older Canadians using data from the CLSA. We found similar proportions of participants who reported increased (13%) and decreased (13.3%) alcohol intake. Factors associated with a change in alcohol intake (increase or decrease) since the start of the pandemic were younger age, higher education and income, living alone, previous and current cannabis use, depression, anxiety, and both negative and positive views of the consequences of the pandemic.

Sociodemographic characteristics that predicted increased alcohol intake compared to no change included female sex, younger age, higher educational attainment, higher annual household income, and living alone. Other Canadian studies conducted during the COVID-19 pandemic reported that living with others was associated with increased alcohol intake (Shield et al., 2020). However, we found this variable was associated with both increased and decreased consumption. Research suggests that individuals with higher socioeconomic status are more likely to drink and engage in high-risk drinking behaviours, but those with lower socioeconomic status are more likely to experience the adverse effects of risky drinking (Public Health Agency of Canada, 2016). Factors that modify the effects of at-risk alcohol consumption include age, sex, gender, race, ethnicity, neighbourhood residence, and housing (Collins, 2016). While the prevalence of heavy episodic drinking is greater among higher-income groups, individuals from lower-income groups report greater frequency and quantity consumed (Centers for Disease Control and Prevention, 2012). We cannot comment on the health consequences of the increased intake we found and whether they were modified by education and income. This would be an important area for further inquiry.

European studies conducted during the first wave of COVID-19 found relationships between increased alcohol consumption and stress from confinement, working from home, living in an urban setting (Reynolds et al., 2021), female sex, lower socioeconomic status (Jackson et al., 2021), younger age (18–49 years), parents of children under 16 years of age, and increasing numbers of children at home (Guignard et al., 2021; Vanderbruggen et al., 2020). Relative to our work, these studies were smaller, enrolled younger participants, and dealt with a more restricted time frame.

Cannabis use (especially in females), mental health concerns, and views on the personal consequences of COVID-19 were associated with increased alcohol intake. Anxiety and depression have previously been found to be associated with increased intake (Anker, 2019; Guignard et al., 2021; Public Health Agency of Canada, 2016). Their relationship is complex. Anxiety may lead to alcohol use, alcohol use may contribute to anxiety, or both may be related to a common third factor (Anker, 2019).

Male sex and living alone were associated with decreased alcohol intake. Several factors were associated with both increased and decreased intake compared to no change, but in all comparisons, the magnitude of the association was greater for increased intake. These included education, income, current cannabis use, depression in females, and both negative and positive consequences of COVID-19. Anxiety was not associated with decreased alcohol intake. The finding that some factors were associated with both increased intake as well as decreased intake may seem contradictory; however, it may reflect individual differences in responses to external stressors. Some individuals may consume alcohol more frequently to cope with stress while others may consume it less in an effort to maintain emotional stability or a sense of control, or to optimize health. The pandemic and our collective response, including public health measures, may have resulted in loss of income and reduced opportunities to socialize, leading to decreased intake for some participants. A United Kingdom study found that decreased alcohol intake during the pandemic was associated with low income and adhering to COVID-19 public health measures, in addition to other factors (Garnett et al., 2021).

Binge drinking has been linked to unintentional injuries and chronic conditions (e.g. high blood pressure, stroke, heart and liver disease, certain cancers, memory difficulties) (World Health Organization, 2018). In our study, 11% of individuals met criteria for occasional and 17% met criteria for regular binge drinking. Participants who were male, were < 65 years of age, had higher education and income, previously or currently used cannabis (especially females), or were female with anxiety and/or depression were more likely to meet the criterion for binge drinking. At CLSA baseline (2011–2015), a higher percentage met criteria for occasional (22.2%) or regular (17.6%) binge drinking than those who participated in the COVID-19 exit survey (11% occasional and 17% regular binge drinking) (Davison et al., 2020). The lower percentages during the more recent data collection may be due to preferential attrition of participants with less healthy lifestyles or factors directly related to the pandemic such as access or income barriers. In the present analysis, participants from Newfoundland, Nova Scotia, and Quebec and females from Prince Edward Island had greater odds of binge drinking than participants from Ontario. Statistics Canada reported in 2021 that residents from the first 3 provinces (i.e. excluding P.E.I.) had higher rates of binge drinking than the Canadian average, based on the same definition of binge drinking we used in our study (Statistics Canada, 2021b).

A relatively unexplored area is the effect of community prevalence of COVID-19 and related provincial public health restrictions on changes in alcohol use. Alcohol sales were deemed an essential service in all provinces and territories during the pandemic (Canadian Centre on Substance Use and Addiction, 2021b). The influence of factors that may have varied across provinces such as the ability of restaurants to sell alcohol for pick up or delivery, alcohol delivery from liquor stores, reduced minimum pricing of alcohol, and permitting consumption in outdoor areas is not known (Canadian Centre on Substance Use and Addiction, 2021b). Quebec residents were more likely than those from any other province to engage in occasional (12%) or regular (20%) binge drinking during the first 10 months of the pandemic. Quebec’s experience with COVID-19 during the first wave was notably challenging (Vogel & Eggertson, 2020). This may have contributed to the greater occurrence of binge drinking during the study time period.

### Strengths and limitations

The CLSA is a large, nationally representative Canadian study with extensive pre-COVID-19 data available on participants. Investigators quickly pivoted data collection at the onset of the pandemic to collect real-time data on its impacts on individuals that could be linked to data collected prior to the start of the pandemic.

A main limitation of the study is the self-reported nature of the responses with the potential for both recall and social desirability bias. The definition of cannabis use was restricted to smoking cannabis and did not include oral or topical use, which may have led to an underestimation of the true frequency of cannabis use. Female sex, older age, lower education, living alone, depression, anxiety, loneliness, and the consequences of the pandemic were associated with missing data, which may have introduced bias into the estimates. The seasonal variation in alcohol consumption is another potential bias and may have arisen by our limited time frame for data collection, which corresponded to the spring and summer of 2020 (Lemmens & Knibbe, 1993; Uitenbroek, 1996). Finally, CLSA participants tend to be highly educated and predominantly urban dwelling (Raina et al., 2019), which may lead to over-representation of certain groups compared with the greater Canadian population.

## Contributions to knowledge

What does this study add to existing knowledge?

• Thirteen percent of middle-aged and older CLSA participants increased their alcohol intake during the first 2 waves of the pandemic, 13.3% decreased their alcohol intake, and 28% met criteria for either occasional or regular binge drinking.

• Predictors of increased alcohol intake included female sex, younger age, education, income, living alone, cannabis use, and mental health concerns.

• Predictors of decreased alcohol intake included male sex and living alone.

• Predictors of binge drinking included male sex, younger age, education, income, cannabis use, and poor mental health in females.

What are the key implications for public health interventions, practice, or policy?

• Specific subpopulations were at greater risk of increasing their alcohol intake or meeting criteria for binge drinking during the COVID-19 pandemic. Their characteristics include younger ages, higher education and income, living alone, use of cannabis, and presence of anxiety or depression.

• Public health interventions (e.g. public awareness campaigns, resources for at-risk groups, educational programs for their health care providers) for reducing harm from at-risk alcohol use could be targeted to individuals with one or more of these characteristics.

## Supplementary Information

ESM 1 (DOCX 74 kb)

## Data Availability

Data are available from the Canadian Longitudinal Study on Aging (www.clsa-elcv.ca) for researchers who meet the criteria for access to de-identified CLSA data.

## Code availability

Code can be made available upon request from the corresponding author.
