# Supplementary material for: Predictors of reported alcohol intake during the first and second waves of the COVID-19 pandemic in Canada among middle-aged and older adults: results from the Canadian Longitudinal Study on Aging (CLSA)
Source: Can J Public Health. 2022 Jul 11;113(5):665–77. doi: 10.17269/s41997-022-00661-5 (PMC9273697; doi:10.17269/s41997-022-00661-5)
Supplement: Supplementary file 1 — (DOCX 74 kb) [file 41997_2022_661_MOESM1_ESM.docx]

**Supplementary Table 1: Descriptive comparison of complete cases and excluding cases for sociodemographic and predictor variables**

|  | Complete cases | | | | Any missing covariates | | | | ρ-value for Total |
| --- | --- | --- | --- | --- | --- | --- | --- | --- | --- |
|  | Increased | Decreased | Stayed the same | Total | Increased | Decreased | Stayed the same | Total |  |
| Total | 2450 | 2498 | 13,901 | 18,849 | 297 | 443 | 2409 | 3149 |  |
|  | 13.0 | 13.3 | 73.7 | 100.0 | 9.4 | 14.1 | 76.5 | 100.0 |  |
| Sex at baseline |  |  |  |  |  |  |  |  |  |
| Male | 1119 | 1309 | 6892 | 9320 | 105 | 201 | 887 | 1193 | <0.0001 |
|  | 12.0 | 14.0 | 73.9 | 100.0 | 8.8 | 16.8 | 74.4 | 100.0 |  |
| Female | 1331 | 1189 | 7009 | 9529 | 192 | 242 | 1522 | 1956 |  |
|  | 14.0 | 12.5 | 73.6 | 100.0 | 9.8 | 12.4 | 77.8 | 100.0 |  |
| 3-level age group at COVID exit |  |  |  |  |  |  |  |  |  |
| <65 | 1275 | 796 | 4552 | 6623 | 125 | 114 | 651 | 890 | <0.0001 |
|  | 19.3 | 12.0 | 68.7 | 100.0 | 14.0 | 12.8 | 73.1 | 100.0 |  |
| 65-74 | 904 | 949 | 5264 | 7117 | 126 | 147 | 771 | 1044 |  |
|  | 12.7 | 13.3 | 74.0 | 100.0 | 12.1 | 14.1 | 73.9 | 100.0 |  |
| 75+ | 271 | 753 | 4085 | 5109 | 46 | 182 | 987 | 1215 |  |
|  | 5.3 | 14.7 | 80.0 | 100.0 | 3.8 | 15.0 | 81.2 | 100.0 |  |
| Province of residence at COVID |  |  |  |  |  |  |  |  |  |
| Newfoundland | 91 | 139 | 840 | 1070 | 7 | 23 | 137 | 167 | <0.0001 |
|  | 8.5 | 13.0 | 78.5 | 100.0 | 4.2 | 13.8 | 82.0 | 100.0 |  |
| Prince Edward Island | 24 | 26 | 237 | 287 | 3 | 9 | 40 | 52 |  |
|  | 8.4 | 9.1 | 82.6 | 100.0 | 5.8 | 17.3 | 76.9 | 100.0 |  |
| Nova Scotia | 201 | 202 | 1278 | 1681 | 20 | 34 | 221 | 275 |  |
|  | 12.0 | 12.0 | 76.0 | 100.0 | 7.3 | 12.4 | 80.4 | 100.0 |  |
| New Brunswick | 26 | 45 | 287 | 358 | 3 | 6 | 36 | 45 |  |
|  | 7.3 | 12.6 | 80.2 | 100.0 | 6.7 | 13.3 | 80.0 | 100.0 |  |
| Quebec | 413 | 415 | 2530 | 3358 | 55 | 77 | 452 | 584 |  |
|  | 12.3 | 12.4 | 75.3 | 100.0 | 9.4 | 13.2 | 77.4 | 100.0 |  |
| Ontario | 645 | 643 | 3171 | 4459 | 65 | 103 | 529 | 697 |  |
|  | 14.5 | 14.4 | 71.1 | 100.0 | 9.3 | 14.8 | 75.9 | 100.0 |  |
| Manitoba | 204 | 242 | 1186 | 1632 | 41 | 47 | 269 | 357 |  |
|  | 12.5 | 14.8 | 72.7 | 100.0 | 11.5 | 13.2 | 75.4 | 100.0 |  |
| Saskatchewan | 27 | 56 | 328 | 411 | 4 | 10 | 79 | 93 |  |
|  | 6.6 | 13.6 | 79.8 | 100.0 | 4.3 | 10.8 | 84.9 | 100.0 |  |
| Alberta | 235 | 239 | 1360 | 1834 | 37 | 61 | 263 | 361 |  |
|  | 12.8 | 13.0 | 74.2 | 100.0 | 10.2 | 16.9 | 72.9 | 100.0 |  |
| British Columbia | 584 | 491 | 2684 | 3759 | 62 | 73 | 383 | 518 |  |
|  | 15.5 | 13.1 | 71.4 | 100.0 | 12.0 | 14.1 | 73.9 | 100.0 |  |
| 4-level education at baseline |  |  |  |  |  |  |  |  |  |
| Less than secondary school | 31 | 85 | 606 | 722 | 2 | 26 | 160 | 188 | <0.0001 |
|  | 4.3 | 11.8 | 83.9 | 100.0 | 1.1 | 13.8 | 85.1 | 100.0 |  |
| Secondary school graduation | 169 | 234 | 1334 | 1737 | 24 | 42 | 301 | 367 |  |
|  | 9.7 | 13.5 | 76.8 | 100.0 | 6.5 | 11.4 | 82.0 | 100.0 |  |
| Some post-secondary education | 142 | 215 | 1001 | 1358 | 15 | 40 | 160 | 215 |  |
|  | 10.5 | 15.8 | 73.7 | 100.0 | 7.0 | 18.6 | 74.4 | 100.0 |  |
| Post-secondary degree/diploma | 2108 | 1964 | 10,960 | 15,032 | 255 | 332 | 1759 | 2346 |  |
|  | 14.0 | 13.1 | 72.9 | 100.0 | 10.9 | 14.2 | 75.0 | 100.0 |  |
| 5-level annual household income at follow-up1 |  |  |  |  |  |  |  |  |  |
| Less than $20,000 | 44 | 80 | 496 | 620 | 2 | 9 | 65 | 76 | <0.0001 |
|  | 7.1 | 12.9 | 80.0 | 100.0 | 2.6 | 11.8 | 85.5 | 100.0 |  |
| $20,000 - $49,999 | 287 | 521 | 2890 | 3698 | 44 | 70 | 422 | 536 |  |
|  | 7.8 | 14.1 | 78.2 | 100.0 | 8.2 | 13.1 | 78.7 | 100.0 |  |
| $50,000-$99,999 | 786 | 968 | 5383 | 7137 | 74 | 121 | 552 | 747 |  |
|  | 11.0 | 13.6 | 75.4 | 100.0 | 9.9 | 16.2 | 73.9 | 100.0 |  |
| $100,000-$149,999 | 650 | 505 | 2891 | 4046 | 38 | 45 | 197 | 280 |  |
|  | 16.1 | 12.5 | 71.5 | 100.0 | 13.6 | 16.1 | 70.4 | 100.0 |  |
| $150,000 or more | 683 | 424 | 2241 | 3348 | 33 | 31 | 170 | 234 |  |
|  | 20.4 | 12.7 | 66.9 | 100.0 | 14.1 | 13.2 | 72.6 | 100.0 |  |
| Number living in household at COVID baseline |  |  |  |  |  |  |  |  |  |
| One (living alone) | 480 | 658 | 3296 | 4434 | 68 | 126 | 658 | 852 | <0.0001 |
|  | 10.8 | 14.8 | 74.3 | 100.0 | 8.0 | 14.8 | 77.2 | 100.0 |  |
| Two | 1375 | 1369 | 8029 | 10,773 | 148 | 205 | 1115 | 1468 |  |
|  | 12.8 | 12.7 | 74.5 | 100.0 | 10.1 | 14.0 | 76.0 | 100.0 |  |
| Three or more | 595 | 471 | 2576 | 3642 | 45 | 59 | 332 | 436 |  |
|  | 16.3 | 12.9 | 70.7 | 100.0 | 10.3 | 13.5 | 76.1 | 100.0 |  |
| Smoked cannabis one month or longer |  |  |  |  |  |  |  |  |  |
| Never | 1853 | 2081 | 11,932 | 15,866 | 214 | 347 | 1987 | 2548 | <0.0001 |
|  | 11.7 | 13.1 | 75.2 | 100.0 | 8.4 | 13.6 | 78.0 | 100.0 |  |
| Previous | 346 | 254 | 1300 | 1900 | 33 | 29 | 157 | 219 |  |
|  | 18.2 | 13.4 | 68.4 | 100.0 | 15.1 | 13.2 | 71.7 | 100.0 |  |
| Current | 251 | 163 | 669 | 1083 | 13 | 18 | 84 | 115 |  |
|  |  |  |  |  |  |  |  |  |  |
| CES-D 10: Screen for depression result |  |  |  |  |  |  |  |  |  |
| Negative screen for depression | 1633 | 1906 | 11,334 | 14,873 | 175 | 278 | 1696 | 2149 | <0.0001 |
|  | 11.0 | 12.8 | 76.2 | 100.0 | 8.1 | 12.9 | 78.9 | 100.0 |  |
| Positive screen for depression | 817 | 592 | 2567 | 3976 | 108 | 139 | 567 | 814 |  |
|  | 20.5 | 14.9 | 64.6 | 100.0 | 13.3 | 17.1 | 69.7 | 100.0 |  |
| GAD-7 Anxiety Severity Classification |  |  |  |  |  |  |  |  |  |
| No/minimal anxiety | 1602 | 1890 | 11,131 | 14,623 | 147 | 249 | 1481 | 1877 | 0.007 |
|  | 11.0 | 12.9 | 76.1 | 100.0 | 7.8 | 13.3 | 78.9 | 100.0 |  |
| Mild anxiety | 620 | 466 | 2225 | 3311 | 55 | 69 | 318 | 442 |  |
|  | 18.7 | 14.1 | 67.2 | 100.0 | 12.4 | 15.6 | 71.9 | 100.0 |  |
| Moderate anxiety | 163 | 103 | 407 | 673 | 24 | 16 | 83 | 123 |  |
|  | 24.2 | 15.3 | 60.5 | 100.0 | 19.5 | 13.0 | 67.5 | 100.0 |  |
| Severe anxiety | 65 | 39 | 138 | 242 | 4 | 7 | 34 | 45 |  |
|  | 26.9 | 16.1 | 57.0 | 100.0 | 8.9 | 15.6 | 75.6 | 100.0 |  |
| UCLA Loneliness Scale- total score |  |  |  |  |  |  |  |  |  |
| Negative | 1769 | 1911 | 11,135 | 14,815 | 167 | 288 | 1597 | 2052 | <0.0001 |
|  | 11.9 | 12.9 | 75.2 | 100.0 | 8.1 | 14.0 | 77.8 | 100.0 |  |
| Positive | 681 | 587 | 2766 | 4034 | 103 | 107 | 567 | 777 |  |
|  | 16.9 | 14.6 | 68.6 | 100.0 | 13.3 | 13.8 | 73.0 | 100.0 |  |
| Consequences of COVID-19 on self and household |  |  |  |  |  |  |  |  |  |
| Very negative | 178 | 136 | 560 | 874 | 18 | 27 | 105 | 150 | <0.0001 |
|  | 20.4 | 15.6 | 64.1 | 100.0 | 12.0 | 18.0 | 70.0 | 100.0 |  |
| Negative | 1628 | 1524 | 7935 | 11,087 | 169 | 212 | 1035 | 1416 |  |
|  | 14.7 | 13.7 | 71.6 | 100.0 | 11.9 | 15.0 | 73.1 | 100.0 |  |
| No effect | 469 | 648 | 4538 | 5655 | 36 | 95 | 596 | 727 |  |
|  | 8.3 | 11.5 | 80.2 | 100.0 | 5.0 | 13.1 | 82.0 | 100.0 |  |
| Positive | 160 | 173 | 810 | 1143 | 16 | 20 | 109 | 145 |  |
|  | 14.0 | 15.1 | 70.9 | 100.0 | 11.0 | 13.8 | 75.2 | 100.0 |  |
| Very positive | 15 | 17 | 58 | 90 | 0 | 3 | 10 | 13 |  |
|  | 16.7 | 18.9 | 64.4 | 100.0 | 0.0 | 23.1 | 76.9 | 100.0 |  |

**Supplementary Table 2: Association of selected predictors with changes in alcohol intake (any changes and either increased or decreased) compared to no changes, mutually adjusted core model and core model adjusted predictors presented with Odds Ratios (OR) along with 95% Confidence Interval (95% CI) in CLSA participants who completed the CLSA COVID-19 exit survey (Fall 2020)**

|  | Any change | Increased | Decreased |
| --- | --- | --- | --- |
| **Core model** | OR (95% CI) | OR (95% CI) | OR (95% CI) |
| **Sex at baseline** |  |  |  |
| Male | 0.97 (0.91,1.04) | 0.84 (0.77,0.92) | 1.12 (1.03,1.22) |
| Female | Ref |  |  |
| 3-level age group at COVID exit |  |  |  |
| <65 | 1.62 (1.48,1.78) | 3.36 (2.91,3.89) | 0.95 (0.85,1.07) |
| 65-74 | 1.34 (1.23,1.47) | 2.36 (2.04,2.72) | 0.98 (0.88,1.09) |
| 75+ | Ref |  |  |
| Province of residence at COVID |  |  |  |
| Newfoundland | 0.67 (0.57,0.78) | 0.51 (0.40,0.65) | 0.82 (0.67,1.00) |
| Prince Edward Island | 0.54 (0.39,0.74) | 0.53 (0.35,0.82) | 0.54 (0.36,0.82) |
| Nova Scotia | 0.79 (0.69,0.90) | 0.79 (0.67,0.95) | 0.78 (0.66,0.93) |
| New Brunswick | 0.64 (0.49,0.83) | 0.48 (0.32,0.73) | 0.78 (0.56,1.08) |
| Quebec | 0.84 (0.76,0.94) | 0.87 (0.75,1.00) | 0.82 (0.72,0.94) |
| Ontario | Ref |  |  |
| Manitoba | 0.94 (0.82,1.06) | 0.85 (0.72,1.02) | 1.01 (0.86,1.19) |
| Saskatchewan | 0.62 (0.48,0.80) | 0.40 (0.26,0.59) | 0.85 (0.63,1.14) |
| Alberta | 0.84 (0.74,0.95) | 0.82 (0.69,0.96) | 0.86 (0.74,1.02) |
| British Columbia | 1.00 (0.91,1.10) | 1.11 (0.98,1.26) | 0.90 (0.79,1.02) |
| 4-level education at baseline |  |  |  |
| Less than secondary school | Ref |  |  |
| Secondary school graduation | 1.33 (1.06,1.68) | 1.68 (1.12,2.51) | 1.26 (0.96,1.64) |
| Some post-secondary education | 1.51 (1.19,1.91) | 1.74 (1.15,2.62) | 1.51 (1.15,1.99) |
| Post-secondary degree/diploma | 1.49 (1.21,1.84) | 2.11 (1.45,3.06) | 1.28 (1.01,1.63) |
| 5-level annual household income at follow-up1 |  |  |  |
| Less than $20,000 | Ref |  |  |
| $20,000 - $49,999 | 1.11 (0.89,1.37) | 1.13 (0.81,1.59) | 1.07 (0.83,1.38) |
| $50,000-$99,999 | 1.19 (0.96,1.46) | 1.43 (1.04,1.98) | 1.03 (0.80,1.33) |
| $100,000-$149,999 | 1.33 (1.07,1.64) | 1.85 (1.34,2.57) | 0.99 (0.76,1.28) |
| $150,000 or more | 1.54 (1.24,1.92) | 2.24 (1.61,3.12) | 1.07 (0.82,1.40) |
| **Core model adjusted** |  |  |  |
|  |  |  |  |
| One (living alone) | 1.22 (1.12,1.33) | 1.22 (1.09,1.38) | 1.22 (1.10,1.36) |
| Two or more | Ref |  |  |
| Smoked cannabis one month or longer | | | |
| Never | Ref |  |  |
| Previous | 1.72 (1.50,1.96) | 2.05 (1.74,2.40) | 1.41 (1.17,1.69) |
| Current | 1.36 (1.19,1.56) | 2.01 (1.72,2.35) | 1.39 (1.16,1.66) |
| CES-D 10: Screen for depression result | | | |
| Negative screen for depression | Ref |  |  |
| Positive screen for depression | 1.79 (1.65,1.93) | 2.29 (2.08,2.53) | 1.39 (1.26,1.55) |
| GAD-7 Anxiety Severity Classification | | | |
| No/minimal anxiety | Ref |  |  |
| Mild anxiety | 1.51 (1.39,1.65) | 1.82 (1.64,2.03) | 1.25 (1.12,1.40) |
| Moderate anxiety | 2.02 (1.72,2.37) | 2.56 (2.11,3.12) | 1.54 (1.23,1.92) |
| Severe anxiety | 2.24 (1.73,2.91) | 2.83 (2.08,3.85) | 1.70 (1.19,2.44) |
| UCLA Loneliness Scale- total score |  |  |  |
| Negative | Ref |  |  |
| Positive | 1.43 (1.32,1.55) | 1.65 (1.49,1.83) | 1.25 (1.13,1.39) |
| Consequences of COVID-19 on self and household | | | |
| Negative | 1.58 (1.46,1.70) | 1.91 (1.71,2.13) | 1.34 (1.21,1.48) |
| No effect | Ref |  |  |
| Positive | 1.63 (1.41,1.87) | 1.74 (1.43,2.11) | 1.55 (1.30,1.86) |

**Supplementary Table 3a. Association of selected predictors with changes in alcohol intake among male participants (any changes and either increased or decreased) compared to no changes, mutually adjusted model presented with Odds Ratios (OR) along with 95% Confidence Interval (95% CI) in CLSA participants who completed the CLSA COVID-19 exit survey (Fall 2020)**

| Male | Any change | Increased | Decreased |
| --- | --- | --- | --- |
|  | OR (95% CI) | OR (95% CI) | OR (95% CI) |
| 3-level age group at COVID exit | | | |
| <65 | 1.50 (1.31,1.72) | 2.85 (2.32,3.52) | 0.99 (0.83,1.17) |
| 65-74 | 1.32 (1.17,1.50) | 2.15 (1.75,2.63) | 1.04 (0.90,1.21) |
| 75+ | Ref |  |  |
| Province of residence at COVID | | | |
| Newfoundland | 0.69 (0.55,0.87) | 0.52 (0.37,0.74) | 0.83 (0.63,1.08) |
| Prince Edward Island | 0.56 (0.36,0.87) | 0.39 (0.19,0.82) | 0.69 (0.41,1.15) |
| Nova Scotia | 0.71 (0.59,0.86) | 0.67 (0.51,0.88) | 0.74 (0.59,0.94) |
| New Brunswick | 0.69 (0.48,1.00) | 0.57 (0.32,1.02) | 0.77 (0.49,1.20) |
| Quebec | 0.85 (0.73,0.99) | 0.96 (0.78,1.18) | 0.77 (0.64,0.94) |
| Ontario | Ref |  |  |
| Manitoba | 0.87 (0.72,1.05) | 0.81 (0.62,1.04) | 0.92 (0.73,1.16) |
| Saskatchewan | 0.75 (0.53,1.07) | 0.37 (0.19,0.72) | 1.04 (0.70,1.54) |
| Alberta | 0.81 (0.68,0.97) | 0.79 (0.62,1.01) | 0.83 (0.66,1.04) |
| British Columbia | 0.92 (0.80,1.06) | 1.06 (0.88,1.28) | 0.82 (0.68,0.98) |
| 4-level education at baseline | | | |
| Less than secondary school | Ref |  |  |
| Secondary school graduation | 1.35 (0.96,1.89) | 1.49 (0.82,2.70) | 1.36 (0.92,2.00) |
| Some post-secondary education | 1.66 (1.18,2.34) | 1.99 (1.10,3.60) | 1.60 (1.07,2.37) |
| Post-secondary degree/diploma | 1.45 (1.08,1.97) | 2.00 (1.16,3.43) | 1.28 (0.91,1.81) |
| 5-level annual household income at follow-up1 | | | |
| Less than $20,000 | Ref |  |  |
| $20,000 - $49,999 | 1.41 (0.97,2.05) | 1.51 (0.85,2.71) | 1.30 (0.84,2.02) |
| $50,000-$99,999 | 1.48 (1.03,2.13) | 1.99 (1.13,3.51) | 1.21 (0.79,1.87) |
| $100,000-$149,999 | 1.68 (1.16,2.44) | 2.51 (1.42,4.45) | 1.27 (0.81,1.98) |
| $150,000 or more | 2.02 (1.39,2.94) | 3.28 (1.84,5.82) | 1.39 (0.89,2.18) |
| Number living in household at COVID baseline | | | |
| One (living alone) | 1.23 (1.07,1.41) | 1.26 (1.03,1.53) | 1.22 (1.03,1.45) |
| Two or more | Ref |  |  |
| Smoked cannabis one month or longer | | | |
| Never | Ref |  |  |
| Previous | 1.08 (0.94,1.25) | 1.24 (1.03,1.49) | 0.94 (0.78,1.14) |
| Current | 1.38 (1.16,1.64) | 1.58 (1.28,1.96) | 1.22 (0.97,1.53) |
| CES-D 10: Screen for depression result | | | |
| Negative screen for depression | Ref |  |  |
| Positive screen for depression | 1.41 (1.21,1.65) | 1.69 (1.38,2.07) | 1.20 (0.99,1.47) |
| GAD-7 Anxiety Severity Classification | | | |
| No/minimal anxiety | Ref |  |  |
| Mild anxiety | 1.16 (1.00,1.35) | 1.24 (1.02,1.51) | 1.08 (0.89,1.31) |
| Moderate anxiety | 1.43 (1.07,1.90) | 1.56 (1.09,2.23) | 1.30 (0.89,1.89) |
| Severe anxiety | 1.22 (0.77,1.94) | 1.26 (0.72,2.22) | 1.13 (0.61,2.11) |
| UCLA Loneliness Scale- total score | | | |
| Negative | Ref |  |  |
| Positive | 1.14 (1.00,1.30) | 1.17 (0.98,1.40) | 1.12 (0.95,1.33) |
| Consequences of COVID-19 on self and household | | | |
| Negative | 1.35 (1.21,1.51) | 1.59 (1.35,1.88) | 1.20 (1.05,1.38) |
| No effect | Ref |  |  |
| Positive | 1.59 (1.28,1.97) | 1.72 (1.27,2.33) | 1.51 (1.16,1.97) |

**Supplementary Table 3b. Association of selected predictors with changes in alcohol intake among female participants (any changes and either increased or decreased) compared to no changes, mutually adjusted model presented with Odds Ratios (OR) along with 95% Confidence Interval (95% CI) in CLSA participants who completed the CLSA COVID-19 exit survey (Fall 2020)**

| Female | Any change | Increased | Decreased |
| --- | --- | --- | --- |
|  | OR (95% CI) | OR (95% CI) | OR (95% CI) |
| 3-level age group at COVID exit | | | |
| <65 | 1.37 (1.19,1.58) | 2.84 (2.28,3.53) | 0.81 (0.68,0.97) |
| 65-74 | 1.22 (1.07,1.39) | 2.22 (1.80,2.75) | 0.87 (0.74,1.02) |
| 75+ | Ref |  |  |
| Province of residence at COVID | | | |
| Newfoundland | 0.73 (0.58,0.92) | 0.59 (0.42,0.81) | 0.89 (0.66,1.19) |
| Prince Edward Island | 0.64 (0.41,1.02) | 0.87 (0.50,1.51) | 0.43 (0.21,0.89) |
| Nova Scotia | 0.91 (0.75,1.10) | 0.96 (0.76,1.23) | 0.85 (0.66,1.10) |
| New Brunswick | 0.67 (0.45,1.00) | 0.49 (0.26,0.91) | 0.86 (0.53,1.40) |
| Quebec | 1.00 (0.86,1.17) | 1.02 (0.84,1.24) | 0.98 (0.80,1.19) |
| Ontario | Ref |  |  |
| Manitoba | 0.98 (0.81,1.17) | 0.86 (0.67,1.09) | 1.10 (0.87,1.39) |
| Saskatchewan | 0.55 (0.38,0.79) | 0.43 (0.25,0.72) | 0.68 (0.43,1.09) |
| Alberta | 0.86 (0.72,1.02) | 0.81 (0.65,1.03) | 0.90 (0.71,1.14) |
| British Columbia | 1.05 (0.91,1.20) | 1.10 (0.92,1.31) | 0.99 (0.82,1.19) |
| 4-level education at baseline | | | |
| Less than secondary school | Ref |  |  |
| Secondary school graduation | 1.37 (0.99,1.90) | 1.92 (1.10,3.36) | 1.20 (0.82,1.75) |
| Some post-secondary education | 1.33 (0.95,1.87) | 1.47 (0.82,2.62) | 1.41 (0.96,2.08) |
| Post-secondary degree/diploma | 1.48 (1.10,1.99) | 2.13 (1.26,3.60) | 1.26 (0.90,1.76) |
| 5-level annual household income at follow-up1 | | | |
| Less than $20,000 | Ref |  |  |
| $20,000 - $49,999 | 1.19 (0.91,1.56) | 1.30 (0.85,2.00) | 1.12 (0.81,1.55) |
| $50,000-$99,999 | 1.47 (1.12,1.93) | 1.87 (1.23,2.84) | 1.27 (0.91,1.75) |
| $100,000-$149,999 | 1.76 (1.32,2.35) | 2.74 (1.78,4.22) | 1.17 (0.82,1.67) |
| $150,000 or more | 2.09 (1.56,2.81) | 3.38 (2.18,5.25) | 1.28 (0.88,1.84) |
| Number living in household at COVID baseline | | | |
| One (living alone) | 1.15 (1.02,1.29) | 1.12 (0.95,1.31) | 1.17 (1.01,1.36) |
| Two or more | Ref |  |  |
| Smoked cannabis one month or longer | | | |
| Never | Ref |  |  |
| Previous | 1.44 (1.23,1.70) | 1.50 (1.23,1.84) | 1.39 (1.11,1.74) |
| Current | 2.05 (1.65,2.54) | 2.44 (1.90,3.14) | 1.59 (1.17,2.17) |
| CES-D 10: Screen for depression result | | | |
| Negative screen for depression | Ref |  |  |
| Positive screen for depression | 1.41 (1.24,1.62) | 1.66 (1.39,1.97) | 1.20 (1.00,1.43) |
| GAD-7 Anxiety Severity Classification | | | |
| No/minimal anxiety | Ref |  |  |
| Mild anxiety | 1.21 (1.07,1.38) | 1.31 (1.11,1.54) | 1.11 (0.93,1.33) |
| Moderate anxiety | 1.36 (1.08,1.72) | 1.46 (1.10,1.94) | 1.21 (0.87,1.68) |
| Severe anxiety | 1.60 (1.13,2.26) | 1.67 (1.11,2.52) | 1.46 (0.90,2.36) |
| UCLA Loneliness Scale- total score | | | |
| Negative | Ref |  |  |
| Positive | 1.02 (0.91,1.15) | 1.06 (0.91,1.23) | 1.01 (0.86,1.18) |
| Consequences of COVID-19 on self and household | | | |
| Negative | 1.51 (1.34,1.69) | 1.68 (1.44,1.97) | 1.35 (1.16,1.57) |
| No effect | Ref |  |  |
| Positive | 1.69 (1.40,2.04) | 1.78 (1.38,2.30) | 1.62 (1.27,2.07) |

**Supplementary Table 4. Association of selected predictors with changes in alcohol intake (any changes and either increased or decreased) compared to no changes, mutually adjusted model presented with Odds Ratios (OR) along with 95% Confidence Interval (95% CI) in CLSA participants who completed the CLSA COVID-19 exit survey (Fall 2020). Missing data were replaced with multiple imputation method**

|  | Any change | Increased | Decreased |
| --- | --- | --- | --- |
|  | OR (95% CI) | OR (95% CI) | OR (95% CI) |
| Sex at baseline | | | |
| Male | 1.04 (0.98,1.11) | 0.89 (0.81,0.97) | 1.20 (1.11,1.31) |
| Female | Ref |  |  |
| 3-level age group at COVID exit |  |  |  |
| <65 | 1.44 (1.32,1.57) | 2.92 (2.54,3.35) | 0.91 (0.81,1.01) |
| 65-74 | 1.29 (1.19,1.41) | 2.29 (2.00,2.62) | 0.97 (0.88,1.07) |
| 75+ | Ref |  |  |
| Province of residence at COVID |  |  |  |
| Newfoundland | 0.72 (0.62,0.84) | 0.56 (0.44,0.70) | 0.86 (0.71,1.04) |
| Prince Edward Island | 0.65 (0.49,0.86) | 0.63 (0.42,0.95) | 0.67 (0.46,0.96) |
| Nova Scotia | 0.81 (0.71,0.92) | 0.82 (0.69,0.97) | 0.80 (0.68,0.94) |
| New Brunswick | 0.70 (0.54,0.91) | 0.55 (0.37,0.82) | 0.82 (0.60,1.12) |
| Quebec | 0.94 (0.85,1.03) | 1.00 (0.88,1.15) | 0.88 (0.77,1.00) |
| Ontario | Ref |  |  |
| Manitoba | 0.93 (0.83,1.05) | 0.87 (0.74,1.02) | 0.98 (0.85,1.14) |
| Saskatchewan | 0.62 (0.49,0.78) | 0.40 (0.27,0.58) | 0.82 (0.62,1.08) |
| Alberta | 0.87 (0.78,0.98) | 0.83 (0.71,0.97) | 0.91 (0.79,1.05) |
| British Columbia | 0.99 (0.90,1.09) | 1.09 (0.97,1.23) | 0.90 (0.80,1.02) |
| 4-level education at baseline |  |  |  |
| Less than secondary school | Ref |  |  |
| Secondary school graduation | 1.31 (1.06,1.62) | 1.92 (1.30,2.83) | 1.17 (0.92,1.49) |
| Some post-secondary education | 1.49 (1.20,1.85) | 1.90 (1.28,2.82) | 1.46 (1.14,1.87) |
| Post-secondary degree/diploma | 1.48 (1.22,1.78) | 2.41 (1.68,3.45) | 1.22 (0.99,1.50) |
| 5-level annual household income at follow-up1 | | | |
| Less than $20,000 | Ref |  |  |
| $20,000 - $49,999 | 1.27 (1.04,1.57) | 1.45 (1.04,2.03) | 1.17 (0.92,1.49) |
| $50,000-$99,999 | 1.48 (1.20,1.81) | 1.96 (1.41,2.71) | 1.24 (0.98,1.58) |
| $100,000-$149,999 | 1.72 (1.39,2.13) | 2.68 (1.92,3.75) | 1.23 (0.96,1.59) |
| $150,000 or more | 2.01 (1.62,2.49) | 3.33 (2.38,4.66) | 1.32 (1.01,1.71) |
| Number living in household at COVID baseline | | | |
| One (living alone) | 1.16 (1.07,1.26) | 1.14 (1.01,1.28) | 1.19 (1.08,1.32) |
| Two or more | Ref |  |  |
| Smoked cannabis one month or longer | | | |
| Never | Ref |  |  |
| Previous | 1.22 (1.10,1.35) | 1.35 (1.18,1.54) | 1.09 (0.95,1.25) |
| Current | 1.57 (1.38,1.79) | 1.85 (1.58,2.16) | 1.33 (1.12,1.58) |
| CES-D 10: Screen for depression result | | | |
| Negative screen for depression | Ref |  |  |
| Positive screen for depression | 1.43 (1.30,1.57) | 1.63 (1.44,1.84) | 1.27 (1.12,1.44) |
| GAD-7 Anxiety Severity Classification | | | |
| No/minimal anxiety | Ref |  |  |
| Mild anxiety | 1.18 (1.07,1.29) | 1.27 (1.13,1.43) | 1.09 (0.96,1.23) |
| Moderate anxiety | 1.35 (1.14,1.60) | 1.50 (1.22,1.85) | 1.16 (0.92,1.46) |
| Severe anxiety | 1.35 (1.04,1.75) | 1.39 (1.01,1.91) | 1.26 (0.89,1.78) |
| UCLA Loneliness Scale- total score |  |  |  |
| Negative | Ref |  |  |
| Positive | 1.05 (0.97,1.14) | 1.11 (0.99,1.24) | 1.01 (0.91,1.13) |
| Consequences of COVID-19 on self and household | | | |
| Negative | 1.41 (1.31,1.53) | 1.66 (1.48,1.85) | 1.25 (1.14,1.38) |
| No effect | Ref |  |  |
| Positive | 1.60 (1.40,1.83) | 1.75 (1.45,2.11) | 1.51 (1.28,1.79) |

**Supplementary Table 5. Association of selected predictors with binge drinking status presented with Odds Ratios (OR) along with 95% Confidence Interval (95% CI) in CLSA participants who completed the CLSA COVID-19 exit survey (Fall 2020). Mutually adjusted model presented for both sexes and stratified by sex. Missing data were replaced with multiple imputation method**

|  | **Both sexes** | **Male** | **Female** |
| --- | --- | --- | --- |
|  | **OR (95% CI)** | **OR (95% CI)** | **OR (95% CI)** |
| **Sex at baseline** | | | |
| Male | 1.27 (1.19,1.36) |  |  |
| Female | Ref |  |  |
| **3-level age group at COVID exit** | | | |
| <65 | 2.88 (2.62,3.16) | 2.71 (2.38,3.07) | 3.12 (2.71,3.61) |
| 65-74 | 1.98 (1.81,2.17) | 1.87 (1.66,2.11) | 2.15 (1.87,2.47) |
| 75+ | Ref |  |  |
| **Province of residence at covid** | | | |
| Newfoundland | 1.30 (1.13,1.51) | 1.32 (1.08,1.61) | 1.29 (1.04,1.59) |
| Prince Edward Island | 1.06 (0.81,1.38) | 0.83 (0.57,1.22) | 1.36 (0.94,1.96) |
| Nova Scotia | 1.27 (1.13,1.44) | 1.32 (1.12,1.56) | 1.22 (1.02,1.45) |
| New Brunswick | 0.86 (0.67,1.11) | 0.87 (0.62,1.23) | 0.85 (0.58,1.24) |
| Quebec | 1.61 (1.46,1.78) | 1.48 (1.29,1.70) | 1.75 (1.52,2.01) |
| Ontario | Ref |  |  |
| Manitoba | 1.04 (0.92,1.18) | 1.04 (0.87,1.24) | 1.05 (0.88,1.25) |
| Saskatchewan | 0.72 (0.57,0.91) | 0.70 (0.49,0.98) | 0.75 (0.54,1.04) |
| Alberta | 0.89 (0.78,1.00) | 0.89 (0.75,1.06) | 0.89 (0.74,1.06) |
| British Columbia | 1.00 (0.90,1.10) | 0.99 (0.86,1.13) | 1.01 (0.88,1.17) |
| **4-level education at baseline** | | | |
| Less than secondary school | Ref |  |  |
| Secondary school graduation | 1.30 (1.04,1.64) | 1.12 (0.85,1.50) | 1.43 (1.06,1.94) |
| Some post-secondary education | 1.26 (0.99,1.59) | 1.09 (0.81,1.47) | 1.39 (1.01,1.91) |
| Post-secondary degree/diploma | 1.03 (0.84,1.27) | 0.91 (0.70,1.17) | 1.22 (0.92,1.61) |
| **5-level annual HH income at follow-up1** | | | |
| Less than $20,000 | Ref |  |  |
| $20,000 - $49,999 | 1.41 (1.11,1.80) | 1.36 (0.94,1.98) | 1.44 (1.08,1.93) |
| $50,000-$99,999 | 2.19 (1.73,2.78) | 2.16 (1.50,3.12) | 2.02 (1.51,2.71) |
| $100,000-$149,999 | 2.61 (2.04,3.34) | 2.45 (1.70,3.55) | 2.68 (1.98,3.63) |
| 150,000 or more | 3.42 (2.67,4.39) | 3.44 (2.37,5.00) | 3.12 (2.29,4.26) |
| **Number living in household at COVID baseline** | | | |
| One (living alone) | 1.08 (0.99,1.19) | 1.14 (1.00,1.30) | 1.12 (1.00,1.26) |
| Two or more | Ref |  |  |
| **Smoked cannabis one month or longer** | | | |
| Never | Ref |  |  |
| Previous | 1.52 (1.37,1.69) | 1.44 (1.26,1.63) | 1.61 (1.38,1.88) |
| Current | 2.93 (2.57,3.34) | 2.65 (2.26,3.11) | 3.25 (2.65,3.98) |
| **CES-D 10: Screen for depression result** | | | |
| Negative screen for depression | Ref |  |  |
| Positive screen for depression | 1.24 (1.11,1.38) | 1.11 (0.96,1.29) | 1.27 (1.11,1.45) |
| **GAD-7 Anxiety Severity Classification** | | | |
| no/minimal anxiety | Ref |  |  |
| mild anxiety | 1.14 (1.03,1.26) | 1.13 (0.98,1.31) | 1.16 (1.02,1.32) |
| moderate anxiety | 1.24 (1.02,1.50) | 1.24 (0.93,1.65) | 1.21 (0.96,1.53) |
| severe anxiety | 1.06 (0.79,1.43) | 1.12 (0.70,1.77) | 1.03 (0.72,1.48) |
| **UCLA Loneliness Scale- total score** | | | |
| Negative | Ref |  |  |
| Positive | 0.86 (0.79,0.95) | 0.90 (0.79,1.03) | 0.86 (0.77,0.97) |
| **Consequences of COVID-19 on self and household** | | | |
| Negative | 0.96 (0.89,1.04) | 0.97 (0.88,1.08) | 0.94 (0.85,1.05) |
| No effect | Ref |  |  |
| Positive | 1.04 (0.90,1.20) | 1.01 (0.82,1.24) | 1.08 (0.90,1.30) |
